# Antimony-Doped Tin Oxide Nanocrystals for Enhanced Photothermal Theragnosis Therapy of Cancers

**DOI:** 10.3389/fbioe.2020.00673

**Published:** 2020-06-24

**Authors:** Zhongjing Lv, Jiafeng Li, Feng Yang, Kun Cao, Qiang Bao, Yuhua Sun, Jian Yuan

**Affiliations:** ^1^Department of Stomatology, The Affiliated Hospital of Xuzhou Medical University, Xuzhou, China; ^2^School of Stomatology, Xuzhou Medical University, Xuzhou, China

**Keywords:** Sb doped SnO_2_ nanocrystals, near-infrared absorption, photothermal agents, CT imaging, photothermal theragnosis therapy

## Abstract

The doped semiconductor nanocrystal with free holes in valence band exhibits strong near-infrared (NIR) local surface plasmon resonance effects, which is essential for photothermal agents. Herein, the hydrophilic Sb doped SnO_2_ nanocrystals were successfully prepared by a simple hydrothermal synthesis method. The doping makes the Sb doped SnO_2_ nanocrystals possessing defect structures. Compared with the un-doped SnO_2_ nanocrystals, Sb doped SnO_2_ nanocrystals exhibit stronger absorption in the NIR region from 500 to 1,100 nm and higher photothermal conversion efficiency (up to 73.6%) which makes the synthesized Sb doped SnO_2_ nanocrystals be used as excellent photothermal agents. Importantly, Sb doped SnO_2_ nanocrystals can efficiently kill cancer cells both *in vitro* and *in vivo* under the irradiation of a 980 nm laser with a power density of 0.6 W cm^–2^. In addition, Sb doped SnO_2_ nanocrystals can also be served as efficient CT imaging agents owing to the large X-ray attenuation coefficient of tin.

## Introduction

Photothermal therapy (PTT) is a technique that uses light absorbers (i.e., photothermal agents) to absorb near-infrared (NIR) laser energy to generate excessive heat to “cook” cancer cells (Yang et al., 2010; Li et al., 2013). It is a potential and effective method to target cancer cells without damaging to surrounding healthy tissues. Nanomaterials currently reported with special optical properties are widely served as photothermal therapeutic agents, mainly including the following categories, namely, organic compound nanomaterials, carbon-based nanomaterials, precious metal nanostructures, and semiconductor nanomaterials (Zhang et al., 2012; Chen et al., 2013, 2014; Yang et al., 2013). Among these, the most widely studied photothermal agents are gold nanostructures which have excellent photothermal conversion effects, but their stability decreases after long-term laser irradiation (Zhang et al., 2012). Therefore, researchers have developed some novel photothermal therapy agents with good photostability. Improving the photothermal conversion efficiency of photothermal agents is essential for the practical application of photothermal therapy. For example, the photothermal conversion efficiency of polypyrrole material is 44.7% when excited by 808 nm laser (Chen et al., 2012), the photothermal efficiency of Cu_7.2_S_4_ nanocrystal is 56.7% when excited by 980 nm laser (Li et al., 2014), and the photothermal efficiency of Cu_2–x_Se nanocrystal is 22.7% when excited by 808 nm laser (Hessel et al., 2011). Generally speaking, a photothermal agent which shows higher photothermal conversion efficiency could cause the same death rate of cancer cells with a shorter laser irradiation time, a lower laser irradiation density, or a lower dose of materials. In contrast, a photothermal agent which has lower photothermal conversion efficiency requires higher agent concentration, longer laser irradiation time, or higher laser power density. To give an example, because Cu_2–x_Se nanocrystals have low photothermal conversion efficiency, the 808 nm laser power density required for them as a photothermal agent is as high as 30 W cm^–2^ (Hessel et al., 2011), which is much higher than the laser power density limitation (0.33 W cm^–2^) of the United States National Standard. In addition, in order to increase the *in vivo* circulation time of nanomaterials, the diameter of intravenously injected nanoparticles should generally be between 10 and 100 nm (Jiang et al., 2008; Choi et al., 2009). However, the diameters of some reported photothermal agents deviate from this size range. For example, CuS superstructure (Tian et al., 2011b), W_18_O_49_ nanowire (Chen et al., 2013), Au nanoshell (Liu et al., 2012), hollow CuS nanoparticle (Dong et al., 2013) and polyaniline (Yang et al., 2011) are all larger than 100 nm; Fe_3_O_4_ @ Cu_2–x_S (Tian et al., 2013) and Ge nanoparticles (Lambert et al., 2007) are smaller below 10 nm, thus limiting their bioapplications. It has been reported that large nanoparticles would be excluded through the reticuloendothelial tissue system (principally from the spleen and liver), small nanoparticles may be excluded through the kidney (Osaki et al., 2004; De Jong et al., 2008). In order to meet the extensive needs of PTT in the future, some novel photothermal therapeutic agents showing suitable size, good photostability, high photothermal performance, and low toxicity are necessary to be developed for effective photothermal therapy of cancer cells.

As a new type of photothermal conversion materials, semiconductor nanomaterials have many unique advantages. For example, semiconductor nanomaterials have the advantages of low cost, stable performance, easy functionalization, and facile preparation (Li et al., 2015). Two types of semiconductor photothermal nanomaterials, according to the causes of NIR absorption, have been reported. The first category is defect-structure semiconductor photothermal nanomaterials. The near-infrared absorption for this kind of materials is caused by the migration of carrier concentration caused by defects, and the absorption intensity and position vary with the degree of defects, including copper-based chalcogenides and transition metal oxides (Tian et al., 2011a; Chen et al., 2013). Copper-based chalcogenides are mainly p-type semiconductors with copper defects and many hole carriers whose migration could produce NIR absorption. The near-infrared absorption source of photothermal materials for transition metal oxides is resulted from oxygen deficiency. The second type is intrinsic semiconductors. The near-infrared absorption for this kind of nanomaterials is based on intrinsic band gap absorption (Song et al., 2015), including WS_2_, MoS_2_, Bi_2_Se_3_, etc. Compared with intrinsic semiconductor photothermal conversion materials, there are more types of defect-structured semiconductor photothermal agents. Plasma absorption peaks can also be tuned by defect adjustment to further improving its photothermal effect, but intrinsic semiconductors do not have this property.

The free holes in valence band of doped semiconductor nanocrystal make the nanocrystals strong and tunable near-infrared local surface plasmon resonance effects (LSPRs) (Luther et al., 2011). Furthermore, by doping and adjusting the plasmon resonance wavelength of the nanocrystal to be equal to or close to the wavelength of the driving laser, the photothermal performance of the nanocrystals can be great improved (Li et al., 2014). These properties have prompted us to develop new types of semiconductor nanocrystals with suitable sizes, high self-doping. To the best of our knowledge, this work is the first report on Sn doped SnO_2_ nanocrystals as photothermal agents, with a diameter of approximately 18 nm, strong NIR absorption, 73.6% photothermal conversion efficiency. Most importantly, these nanoparticles can be suitably used as 980 nm laser-driven photothermal therapy agents, and can effectively kill cancer cells both *in vivo* and *in vitro* under the irradiation of a 980 nm laser with a prompt laser power density (0.6 W cm^–2^). In addition, the Sn doped SnO_2_ nanocrystals can also be used as CT imaging agents due to inherent properties, i.e., large X-ray attenuation coefficient of tin.

## Materials and Methods

### Synthesis of Sb Doped SnO_2_ Nanocrystals

For a typical preparation of 10% Sb-doping nanocrystals, SnCl_4_ (0.9 mmol) and SbCl_3_ (0.1 mmol) were dissolved in 5 mL DMF to prepare the precursor. After that, the precursor was added in a Teflon-lined autoclave in a mixed solvent (20 mL DMF and 20 mL ethanol), followed by 0.5 g PVP and 10 mL PEG. The reaction was kept at 160°C for 20 h. The green precipitate was centrifuged at 10,000 rpm for 10 min and washed twice with ethanol. For the synthesis of un-doped SnO_2_ nanocrystals, no SbCl_3_ was added during the process of preparation the precursor. SnCl_4_ (1.0 mmol) was dissolved in 5 mL DMF to prepare the precursor. For the synthesis of varied molar percent Sb-doped SnO_2_ nanocrystals, different molar ratios of SnCl_4_ and SbCl_3_ were added, making the total amount of metal precursors 1 mmol. The following reaction conditions remained unchanged.

### Characterization

For the information of microstructure, morphology, and size of Sb-SnO_2_ nanocrystals and the un-doped SnO_2_ nanocrystals, can be obtained from TEM microscope (JEOL JEM-2010F). XPS was obtained by -ray photoelectron spectrometer (ESCA-Lab). The UV-vis absorption spectrum data is passed through Shimadzu’s UV-1900 UV-vis-NIR spectrophotometer, using a quartz cuvette with a light path of 1 cm. The XRD patterns were achieved from a X-ray diffractometer (Bruker D4). The tin ions can be determined by Leeman laboratory inductively coupled plasma atomic emission spectrometer (ICP-AES).

In order to test the photothermal performance of Sb-SnO_2_ nanocrystals and the un-doped SnO_2_ nanocrystals, a 980 nm laser was used to irradiate through a quartz cuvette filled with nanocrystal dispersions (80 ppm). A 980 nm laser with adjustable external power (0–2 W) was used as the light source. After calibration, the power is ∼0.3 W and the spot size is ∼0.15 cm^2^. Insert a thermocouple with an accuracy of ±0.1°C into the appropriate position in the above aqueous dispersion to avoid direct laser irradiation to the probes. The temperature changes were recorded every 5 s by a thermocouple thermometer.

### CT Imaging With Sb-SnO_2_ Nanocrystals

Sb-SnO_2_ nanocrystals with different concentrations were scanned using a CT scanner. Before injection of Sb-SnO_2_ nanocrystals, the mice were subjected to CT scanning as a control. Then the nanocrystals dispersion (5 mg/kg) was intratumorally injected into tumor model mice, and then the mice were subjected to CT scanning.

### Photothermal Therapy of *in vitro* Cancer Cells With Sb-SnO_2_ Nanocrystals

SCC15 cells were distributed in 96-well plates at a density of 100,000 cells per well, and cultured in RPMI-1640 medium at a temperature of 37°C and a CO_2_ concentration of 5% for 24 h. Subsequently, the cell culture medium was removed, and the cells were washed three times with PBS buffer solution. 100 μL Sb-SnO_2_ nanocrystal dispersed in PBS was added to different wells at a concentration gradient, and the culture was continued for 24 h. Using a 980 nm laser with a power of 0.6 W cm^–2^ (power ∼0.3 W, spot size ∼0.15 cm^2^), the cells were irradiated for 0 min and 5 min, respectively, and then the cell survival rate was detected by CCK-8 essay. All tests are performed independently twice.

### Quantitative Analysis of Extracellular Phagocytosis

The uptake of Sb-SnO_2_ nanocrystals by SCC15 cells by was evaluated by ICP-AES. SCC15 cells were first seeded in 24 well plates, each with a density of 1 × 10^6^ cells. After incubation with for 24 h, 200 μL of Sb-SnO_2_ nanocrystals were then added to different wells (0, 20, 40, and 80 ppm). After incubation for 12 h, the cell culture fluid was removed. Prior to ablation with aqua regia, cells were carefully washed 5 times with PBS, and then diluted with ultrapure water for ICP-AES analysis to measure the amount of nanocrystals taken up by each cell.

### Photothermal Therapy of *in vivo* Cancer Cells With Sb-SnO_2_ Nanocrystals

All animal experiments are conducted according to the guidelines of the Animal Protection and Use Committee. Some immunodeficiency (SCID) nude mice were simultaneously inoculated with 1 × 10^6^ SCC15 cells and the tumors were cultured for 28 days. SCID nude mice were randomly divided into four groups when the tumors grew to 5–8 mm in diameter, In Group 1, the mice were intratumorally injected only with PBS solution (PBS); In Group 2, the mice were only irradiated with 980 nm laser (NIR); In Group 3, the mice were intratumorally injected with un-doped SnO_2_ nanocrystals (80 ppm) dispersed in PBS solution, and then irradiated with 980 nm laser (SnO_2_ + NIR); In Group 4, the mice were intratumorally injected with Sb-SnO_2_ nanocrystals (80 ppm) dispersed in PBS solution, and then irradiated with 980 nm laser (Sb-SnO_2_ + NIR). The tumor mice in the Group 2 and Group 4 were simultaneously irradiated with a 980 nm laser (0.6 W cm^–2^) for 5 min. During laser treatment, real-time infrared thermal imaging of the whole body of the mouse was recorded by using a photothermal analysis medical device attached to an infrared camera.

After the indicated treatments, the mice were sacrificed, and the tumor was removed and embedded in paraffin to make 4 μm slices. These slices were stained with H&E, inspected with the fluorescent lens of the Zeiss lens 40CFL, and the images were processed with the Zeiss image camera system.

### Main Organ Analysis for Long-Term Toxicity

As for the main organ’ histological examination analysis, a healthy mouse was intravenously injected with Sb-SnO_2_ nanocrystals (10 mg/kg); as a control, another mouse was intravenously injected with PBS. After 15 days, the main organs (including heart, kidney, spleen, liver, and lung) from the sacrificed mice were harvested, and then sectioned into 4 μm slices, stained with H&E. The slices were examined via a microscope. To study the distribution of the Sb-SnO_2_ nanocrystals, healthy mice were intravenously injected with Sb-SnO_2_ nanocrystals (10 mg⋅kg^–1^). These mice were sacrificed to extract major organs at indicated time points (i.e., 1, 3, 7, and 14 days, *n* = 4 at each time point). These organs were then solubilized and then diluted using deionized water for ICP-MS analysis to determine tin content in each sample.

## Results and Discussion

**FIGURE 1:**
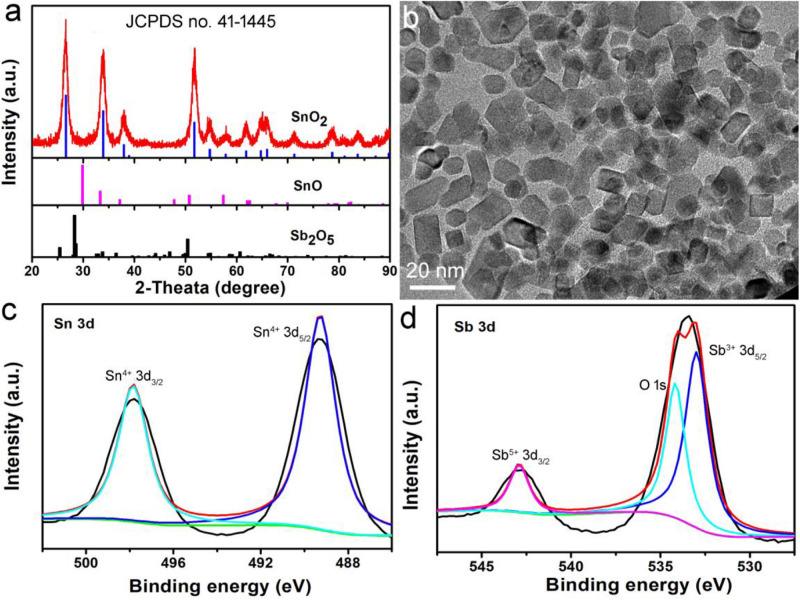
**(a)** XRD patterns and **(b)** TEM image of Sb-SnO_2_ nanocrystals. High resolution XPS spectra of **(c)** Sn 3d and **(d)** Sb 3d in Sb-SnO_2_ nanocrystals.

In the presence of a surface ligand (PVP) and a mixed solvent (ethanol and DMF), hydrophilic nanocrystals coated with PVP (determined by FTIR in Supplementary Figure S1) can be prepared by a simple hydrothermal synthesis method. In order to determine the structural crystal phase of the synthesized nanocrystals, we used X-ray diffractometer to characterize the samples, as shown in Figure 1a. All the X-ray diffraction peaks of the as-prepared products can be well matched with the cassiterite phase SnO_2_ without any other phases (such as Sb_2_O_5_ and SnO), indicating that the doped Sb occurs by replacing Sn atoms in the SnO_2_ structure. In addition, the lattice constants are very close to those in the JCPDS file (No. 14-1445), which proved that the Sb doped SnO_2_ nanocrystals were formed. Transmission electron microscopy (TEM) images show that the Sb-SnO_2_ nanocrystals are well dispersed (Figure 1b), with an average diameter of ∼18 nm. Further microstructural information of the synthesized Sb-SnO_2_ nanocrystals can be obtained from high resolution transmission electron microscopy (HRTEM, Supplementary Figure S2). The HRTEM image shows that the sample is single crystal with a lattice spacing of 0.334 nm, which can be indexed to the (110) crystal plane of the Sb-SnO_2_ crystal. In addition, the obtained fast Fourier transform (FFT) (Supplementary Figure S3) can belonged to the [110] crystal band axis of Sb-SnO_2_ crystals. To further confirming the oxidation states of Sb and Sn atoms, XPS analysis was performed. From the XPS results, one can see that there was only Sb, Sn, and O in the Sb doped SnO_2_ (Sb-SnO_2_) without other impurities (Supplementary Figure S4). The high resolution XPS spectra of Sn 3d in Sb-SnO_2_ were given in Figure 1c. The peaks at 487.4 and 495.8 eV were assigned to Sn 3d_5/2_ and Sn 3d_3/2_ of Sn (IV) in Sb-SnO_2_ crystals, respectively (Xu et al., 2013). The binding energies at 533.4 and 542.8 eV can be, respectively, attributed to Sn^3+^ 3d_5/2_ and Sn^5+^ 3d_3/2_ in Sb-SnO_2_ crystals (Figure 1d). The mixed valence of Sn in Sb-SnO_2_ nanocrystals indicated the defect structures of the nanocrystals, which is essential for the optical properties of semiconductor photothermal agents. The actual doping contents can be obtained from the XPS analysis. It was found to be about 9.2%, which are slightly lower than the target content. Based the results above, it can be proved that we have successfully synthesized Sb-SnO_2_ nanocrystals.

**FIGURE 2:**
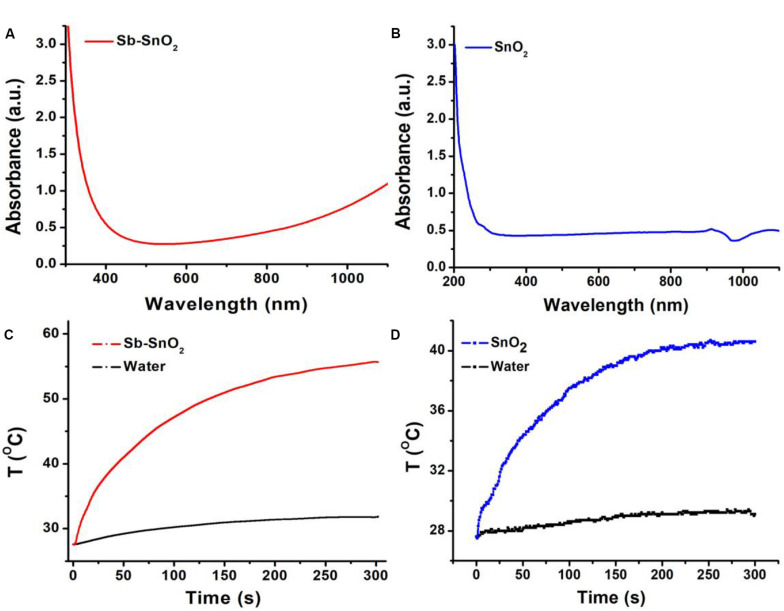
**(A)** UV-vis spectra and **(B)** photothermal effect of Sb-SnO_2_ nanocrystals. **(C)** UV-vis spectra and **(D)** photothermal effect of SnO_2_ nanocrystals.

The most notable feature of the obtained Sb-SnO_2_ nanocrystals is that they have strong absorption in the near infrared region due to the defect structure. PVP-coated Sb-SnO_2_ nanocrystals are well dispersed in water even for a month, still showing strong NIR absorption, indicating that the nanocrystals have good stability and good dispersion. Figure 2A shows the UV-vis-NIR absorption spectra of Sb-SnO_2_ nanocrystals at a concentration of 80 ppm. As demonstrated in Figure 1d, there was defect structure in the Sb-SnO_2_ nanocrystals, which made the nanocrystals showing strong NIR absorption. There was an enhanced absorption from 500 to 1,100 nm. The strong absorption strength is mainly attributed to many defects and high monodispersion. However, the un-doped SnO_2_ nanocrystals showed little NIR absorption (resulted from the band gap absorption) as they have no defect structures (Figure 2B). Due to the near-infrared absorption characteristics of Sb-SnO_2_ nanocrystals and the strong absorption wavelength at 980 nm, these nanocrystals can be better used as photothermal agents for cancer treatment driven by 980 nm laser. We then measured the photothermal performance of Sb-SnO_2_ nanocrystals (80 ppm) under the continuous irradiation of a 980 nm laser with a power of 0.3 W. The nanocrystals’ temperature increased from room temperature (26.7°C) to 57.2°C (Figure 2C). As an alternative, pure water was also irradiated by 980 nm laser for 5 min and only increased from room temperature to 30.2°C, and the temperature rise was less than 4°C. To better illustrate the photothermal effect of Sb-SnO_2_ nanocrystals, the photothermal conversion of un-doped SnO_2_ nanocrystals was also measured. As shown in Figure 2D, the temperature of un-doped SnO_2_ nanocrystals at the same condition only increased from room temperature to 41.8°C which is much lower than that of Sb-SnO_2_ nanocrystals. Therefore, the doping in SnO_2_ nanocrystals made Sb-SnO_2_ nanocrystals possess defect, strong NIR absorption, and excellent photothermal effect.

The photothermal conversion efficiency is an important index for evaluating the photothermal performance of photothermal agents. Generally speaking, photothermal reagents with high photothermal conversion efficiency cause the same death rate of cancer cells only with lower agent concentration, shorter illumination time, or lower laser irradiation power density, which is a healthy biological tissue advantageous. In order to further study the advantages of the photothermal properties of our synthesized Sb-SnO_2_ nanocrystals, we tested the photothermal conversion efficiency of Sb-SnO_2_ nanocrystals.

According to the report of Roper et al. (2007), we tested the photothermal conversion efficiency of Sb-SnO_2_ nanocrystals. The nano-particles are dispersed in the medium (such as water). After laser irradiation with a certain power, the light energy is converted into thermal energy. The heat transferred by the nano-particles to the medium is a fixed value per unit time. When the heat transfer from the nanoparticles to the medium reaches a balance with the heat transfer from the medium to the surrounding environment, the temperature will not change. Based on this, Roper derives the calculation formula (1) of the photothermal conversion efficiency as following:

(1) $$\eta_{\text{T}}\,\text{=}\,\frac{\text{hA}\,\left(T_{\max}-T_{\text{amb}}\right)-Q_{0}}{I\,\left(1-10^{-A_{\lambda }}\right)}$$

In which *h* is the heat transfer coefficient and *A* is the surface area of the container. *T*_max_ is the highest temperature of the system, and *T*_amb_ is the ambient temperature. *I* is the laser power (mW), and *A*_λ_ is the absorption of the medium at the excitation wavelength. *Q*_0_, heat input rate (mW) of the system, can be independently calculated due to the light absorption of the solution. hA can be obtained by measuring the rate of cooling temperature after light source shut off. The value of hA is obtained by the following formula (2):

(2) $$\tau_{\text{s}}=\frac{m_{\text{D}}\,C_{\text{D}}}{h\,A}$$

Where τ_s_ is the time constant of the cooling system after the laser shut off, and *m*_D_ and *C*_D_ are the mass and specific heat capacity of the dispersed nanoparticle medium, respectively.

**FIGURE 3:**
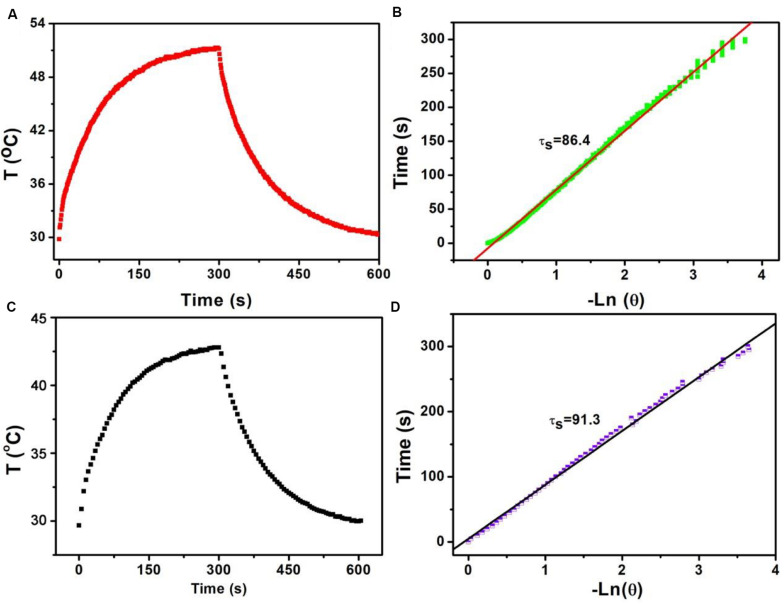
**(A)** Temperature change of Sb-SnO_2_ nanocrystals irradiated by a 980 laser for 300 s, then shut off the laser for 300 s. **(B)** Time constant of Sb-SnO_2_ nanocrystals. **(C)** Temperature change of un-doped SnO_2_ nanocrystals irradiated by a 980 laser for 300 s, then shut off the laser for 300 s. **(D)** Time constant of un-doped SnO_2_ nanocrystals.

Based on the above formula, we can calculate the photothermal conversion efficiency of Sb-SnO_2_ nanocrystals. We used a 980 nm laser with a power density of 0.3 W to irradiate the Sb-SnO_2_ nanocrystal water dispersion (80 ppm), and then the laser was turned off to allow it to cool naturally. The temperature change during the entire process was recorded, as shown in Figure 3A. According to the cooling process after the laser turned off, the negative natural logarithm curve of cooling time and temperature driving force is obtained (Figure 3B). According to Figure 3A, (*T*_max_ − *T*_amb_) is 21.4${}^{{}^{\circ}}$C. According to Figure 2A, the value of *A*_980_ is 0.75. *Q*_0_ was measure to be 130.4 mW by independent measurement of pure water without nanocrystals. The mass of water is 0.3 g and the specific heat capacity is 4.2 J g^–1^. In Figure 3B, the slope of the curve is the time constant of heat transfer in the system, τ_s_ is 86.4 s. Therefore, the heat conversion efficiency of Sb-SnO_2_ nanocrystals under the 980 nm laser irradiation can be calculated to be 73.6%. This value (73.6%) is much higher than those of other semiconductor photothermal agents. To better understanding the effect Sb-doping on photothermal conversion efficiency of Sb- SnO_2_ nanocrystals, we have calculated the photothermal conversion efficiencies of varied Sb-doping. The results were shown in Supplementary Table S1. It was found that photothermal conversion efficiency increased with the increase of Sb doping, but the increasing trend slowed down when the doping content reached to 8%. As a control, the photothermal conversion efficiency of un-doped SnO_2_ nanocrystals under the same conditions was calculated to be 52.4% (Figures 3C,D), much lower than that of Sb-SnO_2_ nanocrystals due to the fact that no defects existed in un-doped SnO_2_ nanocrystals. In general, the photothermal conversion efficiency is higher with higher NIR absorption. In addition, due to the size-dependent light absorption and scattering effects, the photothermal conversion efficiency of Sb-SnO_2_ nanocrystals is higher than those of the previously reported nanoparticles with lager size.

**FIGURE 4:**
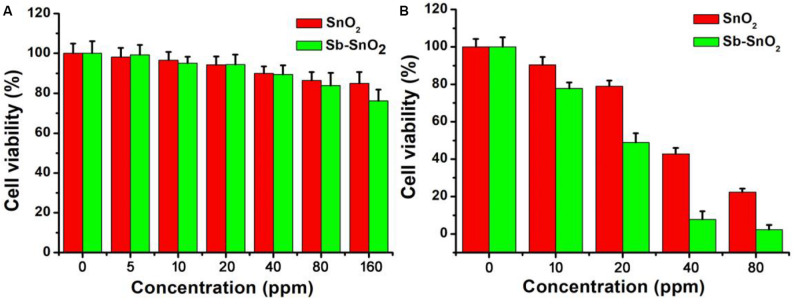
**(A)** CCK-8 essay for Sb-SnO_2_ nanocrystals and SnO_2_ nanocrystals incubated with cells for 24 h, respectively. **(B)** Photothermal therapy of cancer cells *in vitro* with Sb-SnO_2_ nanocrystals and SnO_2_ nanocrystals, respectively.

As the synthesized Sb-SnO_2_ nanocrystals have high photothermal conversion efficiency, we therefore believe that these synthesized nanocrystals can be used as an excellent photothermal therapeutic agent. To confirm our conjecture, we first used the CCK-8 essay with SCC15 cells to evaluate the photothermal toxicity of Sb-SnO_2_ nanocrystals *in vitro*. Therefore, we cultured different concentrations of Sb-SnO_2_ nanocrystal dispersion in PBS (0, 5, 10, 20, 40, 80, and 160 ppm) with SCC15 cells for 24 h, and then tested the cell viability by CCK-8 essay. We first studied the destruction of cells with Sb-SnO_2_ nanocrystalline PBS dispersion in the absence of laser irradiation, as shown in Figure 4A. The results showed that at a material concentration of 40 ppm, the cell survival rate is about 90% (Figure 4A). When the concentration increased to 80 ppm, the cell viability was still above 80%, indicating the good biocompatibility. Compared with the un-doped the SnO_2_ (green histogram), the biocompatibility showed little difference. The good biocompatibility provides an effective reference to evaluate the damage of Sb-SnO_2_ nanocrystals to cells under the laser irradiation. It can be seen from Figure 4B (red histogram) that in the presence of Sb-SnO_2_ nanocrystals (80 ppm), only ∼2% of the cells were survived when irradiated by a 980 nm laser (output power 0.6 W cm^–2^) for 5 min. As a control, ∼98% of the cells without laser irradiation were survived (red histogram), which demonstrated that Sb-SnO_2_ nanocrystals can be of promising photothermal agents. We also evaluated the photothermal toxicity of un-doped SnO_2_ to cancer cells (green histogram). We can directly see that the cell viability is much lower using un-doped SnO_2_ nanocrystals. ∼22% of the cells treated with un-doped SnO_2_ were survived, while only ∼2% of cells survived treated with Sb-SnO_2_ nanocrystals. In order to better evaluate the efficiency of Sb-SnO_2_ nanocrystals as a photothermal agent *in vitro*, it is very necessary to study the uptake effect of nanocrystals by cancer cells. We used ICP-AES to detect the amount of nanocrystals taken by each cell after co-incubation of Sb-SnO_2_ nanocrystals and SCC15 cells for 12 h. As shown in Supplementary Figure S5, with the increase of the concentration of nanocrystals (i.e., 0–80 ppm), the uptake of nanocrystals per cell after 12 h of culture increased (0.080–5.35 pg/cell). This indicated that Sb-SnO_2_ nanocrystals can be engulfed by cells through endocytosis.

**FIGURE 5:**
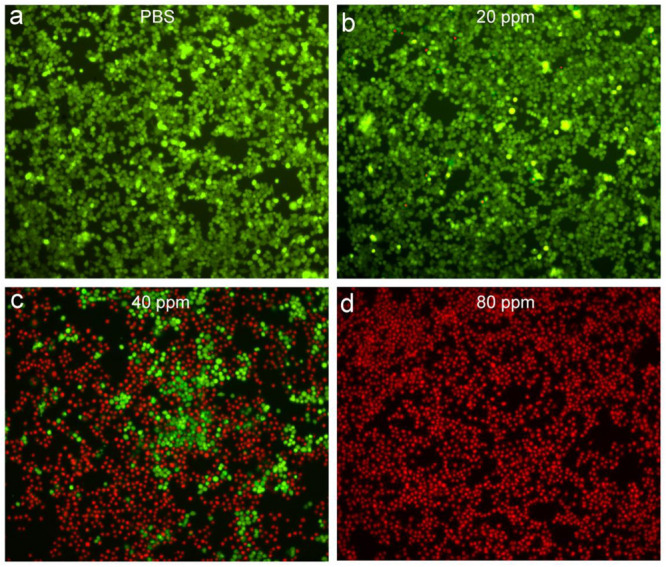
Confocal micrographs of the cells after different treatments: **(a)** PBS, **(b)** 20 ppm nanocrystals, **(c)** 40 ppm nanocrystals, **(d)** 80 ppm nanocrystals. The cells were irradiated by a 980 nm laser with a power density of 0.6 W cm^–2^. Magnification: 100 times.

In order to more intuitively observe the effect of the photothermal effect of Sb-SnO_2_ nanocrystals on the SCC15 cells, we stained the live and dead cells with calcein-AM and propidium iodide. Figures 5a–d shows the confocal micrographs of the cells after different treatments. It indicated that the dead cells increased more with the increase of concentration of nanocrystals. When the concentration of nanocrystals was 80 ppm, all the cells are almost dead. The results of live/dead cell staining analysis were matched well with CCK8 assay. Both the live/dead cell staining analysis and the CCK8 assay confirmed that Sb-SnO_2_ nanocrystals combined with NIR laser irradiation showed a good inhibitory effect on SCC15 cell proliferation.

**FIGURE 6:**
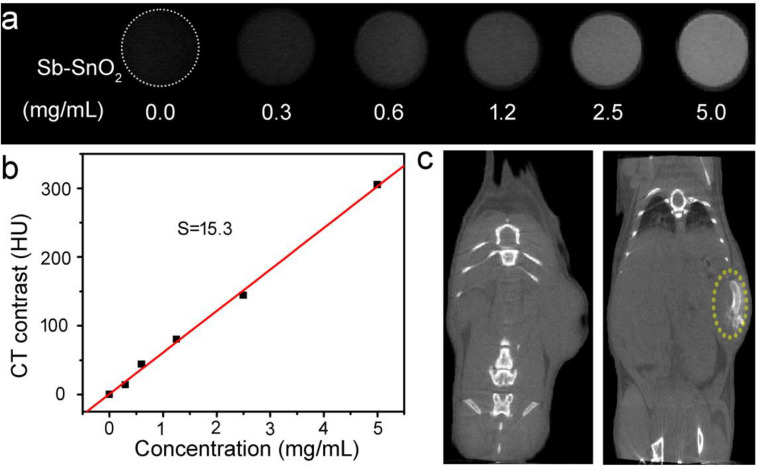
**(a)** CT signals of Sb-SnO_2_ nanocrystals with varied concentrations. **(b)** CT contrast value vs. the concentration of nanocrystals. **(c)** CT image of a mouse before (left) and after (right) the injection of nanocrystals.

Sb-SnO_2_ nanocrystals not only have a good photothermal effect, but also have great potential in CT imaging diagnosis. Since Sn has a large atomic number, it can have a large X-ray attenuation coefficient like Au, Bi, and I (Liu et al., 2016; Zhou et al., 2016). Thus Sb-SnO_2_ nanocrystals can be used as CT contrast agents. To test the hypothesis, we measured the Sb-SnO_2_ nanocrystals with different concentrations for CT imaging scanning experiments. It showed that the CT signal increased with the increase of the concentration of nanocrystals (Figure 6a). At the same time, it can be seen in Figure 6b that as the HU value of nanocrystals increased linearly with concentration, which illustrated the water dispersion of nanocrystals showing good dispersibility. The slope of the HU value of this nanocrystals is about 15.3 HU L/g (Figure 6b), which is high enough for CT imaging. Next, we evaluated the *in vivo* CT imaging performance of Sb-SnO_2_ nanocrystals. Before injection of Sb-SnO_2_ nanocrystals, the mice were subjected to CT scanning as a control. Then the nanocrystals dispersion was injected into tumor model mice by intratumoral injection. Figure 6c reveals the CT view of the mouse tumor site before and after the injection of nanocrystals (100 μL, 5 mg mL^–1^). As shown in the figure, the tumor area has a clear signal after injection compared with before injection, and the corresponding tumor injection site also shows a bright contrast with other soft tissues. At the same time, the CT average of the tumor area is much higher than other soft tissues. These results indicated that Sb-SnO_2_ nanocrystals can be used as an efficiency CT imaging agents.

**FIGURE 7:**
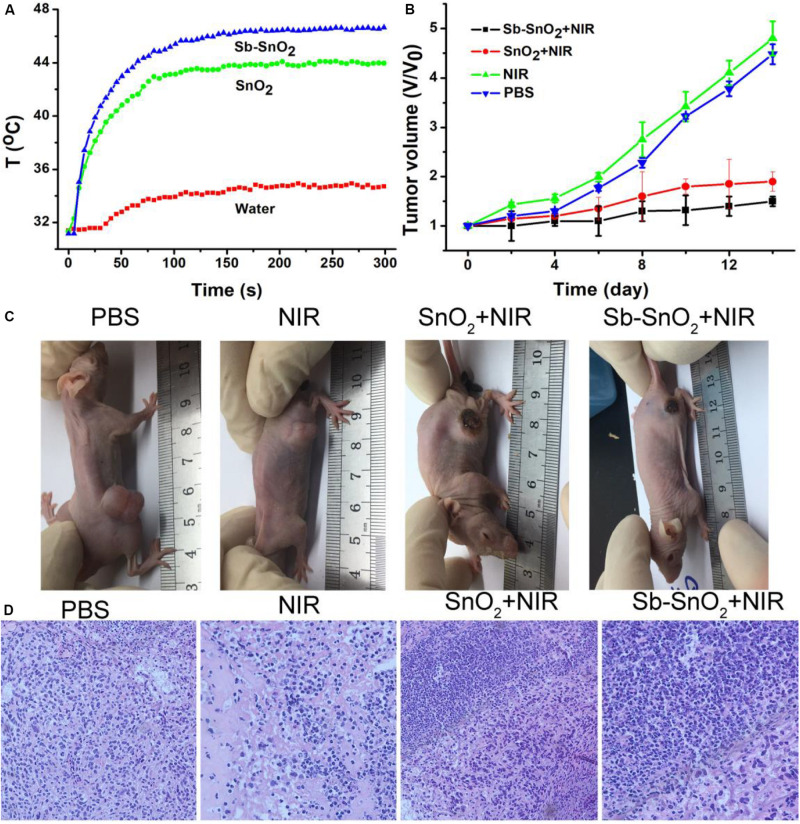
**(A)** Photothermal effect after injection of PBS, SnO_2_, and Sb-SnO_2_, respectively. **(B)** Tumor volume changes in different groups. **(C)** Mice picture after indicated treatments for 14 days. **(D)** H&E images of *ex vivo* tumor sections in different groups.

*In vitro* cell experiments show that Sb-SnO_2_ nanocrystals can effectively kill cancer cells under the drive of 980 nm laser. In order to make an overall assessment of the photothermal effect of Sb-SnO_2_ nanocrystals, we also explored the photothermal treatment effect of Sb-SnO_2_ nanocrystals on tumor model mice driven by 980 nm laser (Supplementary Figure S6). During the laser treatment, an infrared camera can be used to monitor the temperature change the tumor. As shown in Figure 7A, there was a significant heating effect under laser irradiation in Group 4; for comparison, there was a lower temperature increase in Group 3. This happened because Sb-SnO_2_ nanocrystals had better photothermal effect than SnO_2_ nanocrystals. For mice in Group 2, NIR laser alone cannot make the temperature of the tumor obvious increase.

After treatments, tumor volume changes are recorded every 2 days. As shown in Figure 7B, Tumor were significantly suppressed in group 4; the tumor suppression of the third group is lower than that of the fourth group. As comparison, the tumor in Groups 1and 2 grew rapidly and there was no obvious difference between the two groups. We can also see the difference in tumor changes from the pictures of the mice 14 days after treatments (Figure 7C), which was consistent with the tumor growth curves in Figure 7B. These results indicated that Sb-SnO_2_ nanocrystals still showed excellent photothermal performance *in vivo*. It can be concluded that Sb-SnO_2_ nanocrystals combined with NIR laser irradiation can successfully inhibited tumor growth due to the excellent the photothermal effect resulted from Sb-SnO_2_ nanocrystals.

For further evaluation the photothermal ablation effect of tumor cells *in vivo*, we stained the tumor tissue with H&E. The micrograph after staining is shown in Figure 7D. As we expected, a large number of death of cancer cells treated by injection with Sb-SnO_2_ nanocrystals and then laser irradiation were observed, but less cell death in SnO_2_ nanocrystals and laser irradiation. For the control groups, the shape and size of cancer cells were almost unchanged. These results indicated that cancer cells *in vivo* can also be effectively destroyed by the high temperature generated by the photothermal effect of Sb-SnO_2_ nanocrystals. Taken together, these results undoubtedly confirm that the synthesized Sb-SnO_2_ can be used as excellent photothermal theranostics agents due to their excellent photothermal effect and CT imaging performance, and have great potential for photothermal treatment of cancers.

To evaluate the *in vivo* biosafety of Sb-SnO_2_ nanocrystals, further bio-safety experiment on histological examination analysis with H&E staining for the main organs was conducted to observe the size, shape and number of cells after the intravenous injection of Sb-SnO_2_ nanocrystals. From the H&E staining of the major organs including heart, kidney, spleen, liver, and lung, no inflammation or damage is observed (Supplementary Figure S7). To study the distribution of the Sb-SnO_2_ nanocrystals, the contents of nanocrystals accumulated in main organs were also evaluated. It showed (Supplementary Figure S8) that the Sb-SnO_2_ nanocrystals mainly accumulate at liver and spleen, which indicates that this material was mainly degraded in these two organs.

## Conclusion

In conclusion, the hydrophilic Sb doped SnO_2_ nanocrystals with a size of 18 nm were successfully prepared by a facile hydrothermal synthesis method. The doping makes the Sb-SnO_2_ nanocrystals possessing defect structures, which contributes to the enhanced absorption in the NIR region. Thus the Sb-SnO_2_ nanocrystals show excellent photothermal effect, with photothermal conversion efficiency up to 73.6%. Compared with un-doped SnO_2_ nanocrystals, experiments on cancer cells both *in vitro* and *in vivo* proved that the photothermal effect from Sb-SnO_2_ nanocrystals can more effectively kill cancer cells. In addition, Sb-SnO_2_ nanocrystals can also be used as efficient CT imaging agents owing to the large X-ray attenuation coefficient of tin. Therefore, the synthesized Sb-SnO_2_ nanocrystals can be used as excellent photothermal theragnosis agents.

## Data Availability Statement

All datasets generated for this study are included in the article/Supplementary Material.

## Ethics Statement

The animal study was reviewed and approved by Affiliated Hospital of Xuzhou Medical University.

## Author Contributions

ZL and JY designed the project. ZL, JL, FY, and KC carried out the experiments. ZL, QB, and YS performed the experimental data analysis. ZL and JY wrote the manuscript. All authors contributed to discussion of the results.

## Conflict of Interest

The authors declare that the research was conducted in the absence of any commercial or financial relationships that could be construed as a potential conflict of interest.
