# Supplementary material for: Antimony-Doped Tin Oxide Nanocrystals for Enhanced Photothermal Theragnosis Therapy of Cancers
Source: Front Bioeng Biotechnol. 2020 Jun 24;8:673. doi: 10.3389/fbioe.2020.00673 (PMC7358652; doi:10.3389/fbioe.2020.00673)
Supplement: Supplementary file 1 [file Image_1.pdf]

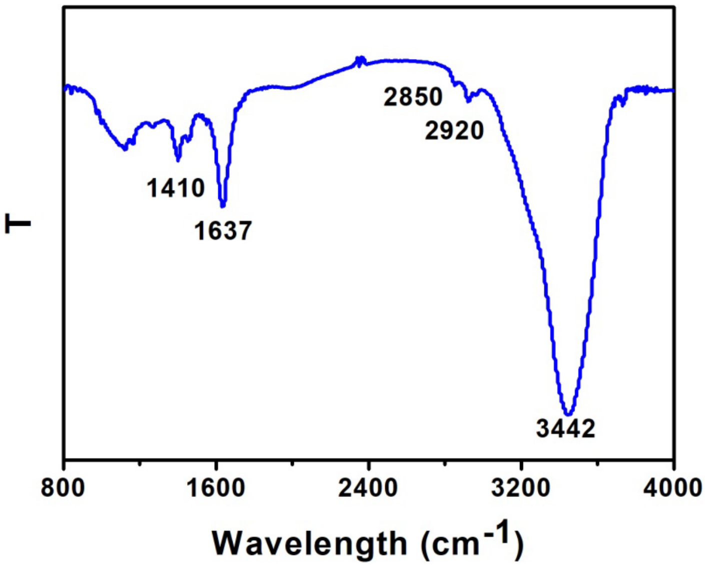

Figure S1. FTIR spectrum of Sb-SnO_2 nanocrystals.

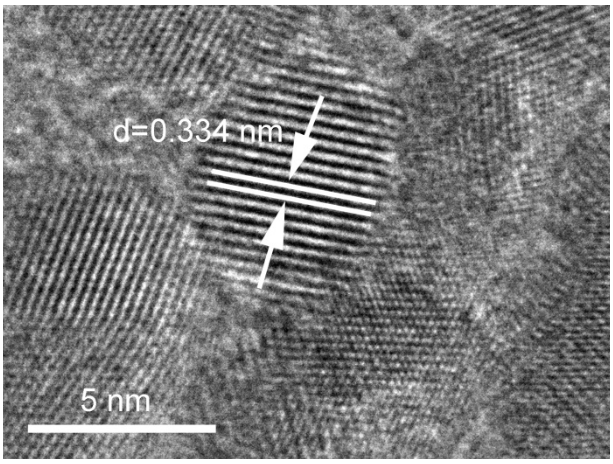

Figure S2. HRTEM image of Sb-SnO_2 nanocrystals.

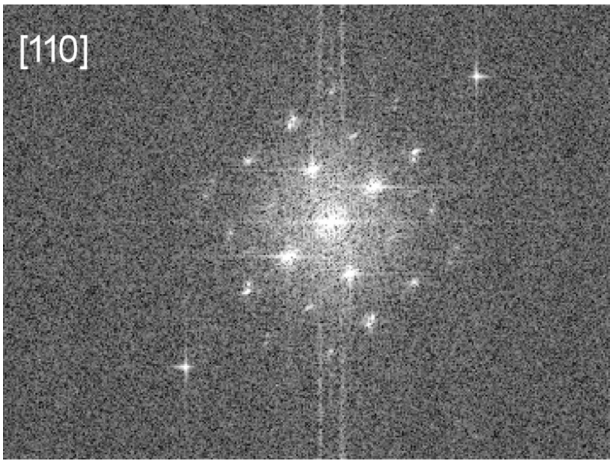

Figure S3. Fast Fourier transform image of Sb-SnO2 nanocrystals obtained from Figure S2.

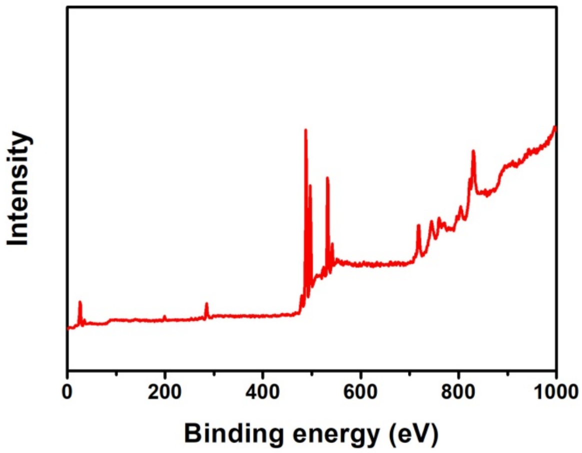

Figure S4. XPS spectra of Sb-SnO2 nanocrystals.

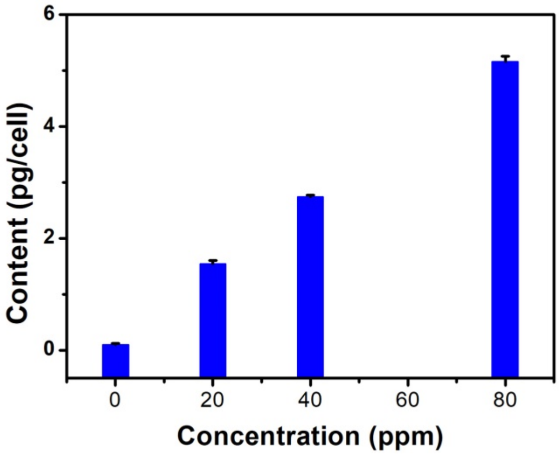

Figure S5. Cellular uptake in vitro of the Sb-SnO_2 nanocrystals with different concentrations.

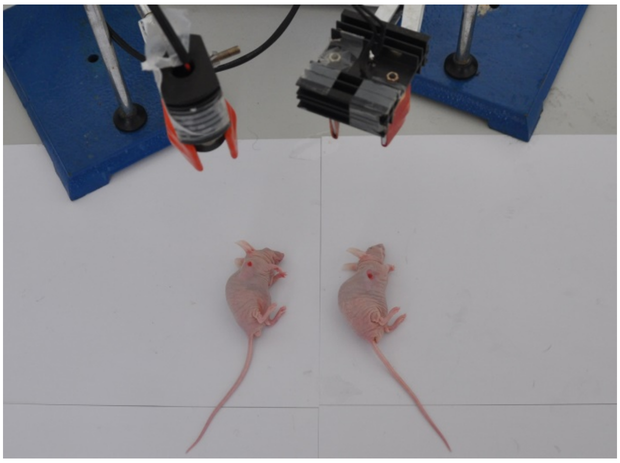

Figure S6. Photograph showing the typical experimental setup for in vivo photothermal therapy of cancer cells.

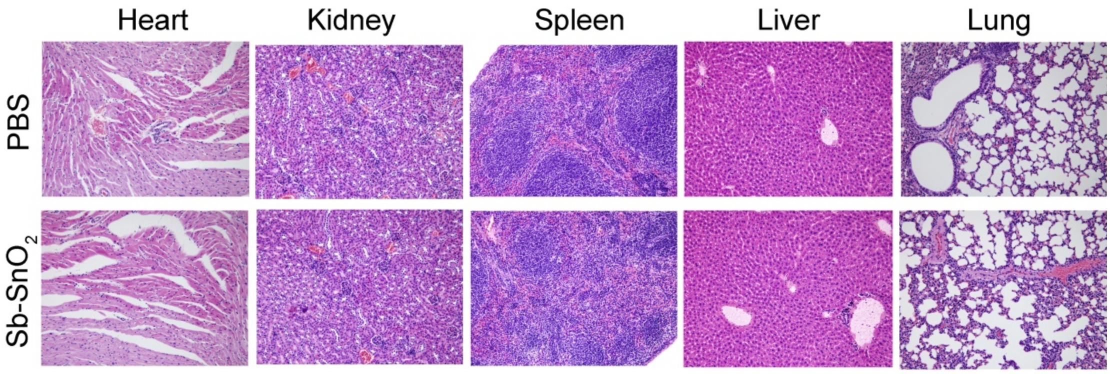

Figure S7. H&E stained slices of main organs. Magnification: 100 times.

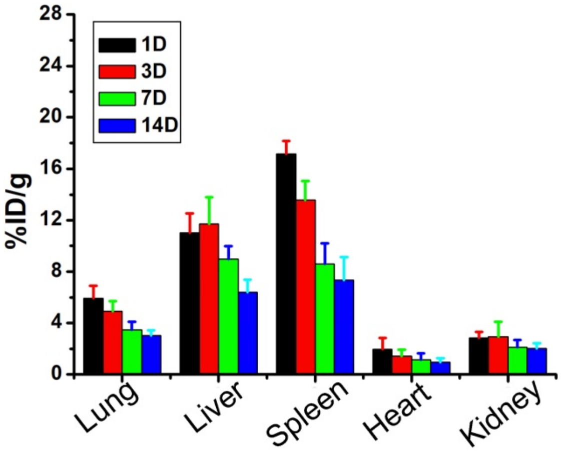

Figure S8. Biodistribution of tin determined by ICP-AES (n = 4) in major organs.
