# Supplementary material for: Antimony-Doped Tin Oxide Nanocrystals for Enhanced Photothermal Theragnosis Therapy of Cancers
Source: Front Bioeng Biotechnol. 2020 Jun 24;8:673. doi: 10.3389/fbioe.2020.00673 (PMC7358652; doi:10.3389/fbioe.2020.00673)
Supplement: Supplementary file 2 [file Table_1.pdf]

Table S1. Effect Sb-doping on photothermal conversion efficiency of Sb-SnO_2 nanocrystals

Doping content (%)	Photothermal conversion efficiency (%)
0	52.4
2	56.9
4	61.3
6	66.3
8	70.2
10	73.6
12	75.1
